# Characterization of Activated Carbons from Oil-Palm Shell by CO_2_ Activation with No Holding Carbonization Temperature

**DOI:** 10.1155/2013/624865

**Published:** 2013-05-07

**Authors:** S. G. Herawan, M. S. Hadi, Md. R. Ayob, A. Putra

**Affiliations:** Faculty of Mechanical Engineering, Universiti Teknikal Malaysia Melaka, Hang Tuah Jaya, 76100 Durian Tunggal, Melaka, Malaysia

## Abstract

Activated carbons can be produced from different precursors, including coals of different ranks, and lignocellulosic materials, by physical or chemical activation processes. The objective of this paper is to characterize oil-palm shells, as a biomass byproduct from palm-oil mills which were converted into activated carbons by nitrogen pyrolysis followed by CO_2_ activation. The effects of no holding peak pyrolysis temperature on the physical characteristics of the activated carbons are studied. The BET surface area of the activated carbon is investigated using N_2_ adsorption at 77 K with selected temperatures of 500, 600, and 700°C. These pyrolysis conditions for preparing the activated carbons are found to yield higher BET surface area at a pyrolysis temperature of 700°C compared to selected commercial activated carbon. The activated carbons thus result in well-developed porosities and predominantly microporosities. By using this activation method, significant improvement can be obtained in the surface characteristics of the activated carbons. Thus this study shows that the preparation time can be shortened while better results of activated carbon can be produced.

## 1. Introduction

Activated carbons are sorbents with a highly developed porosity, especially in the micro and meso ranges, which are used in a wide range of applications, including gas-phase and liquid-phase adsorption processes [1, 2]. Activated carbons can be produced from different precursors, including coals of different ranks, and lignocellulosic materials, by physical or chemical activation processes [2, 3]. In general, the principal properties of manufactured active carbons depend on the type and properties of the raw material used. Recently, increasing attention has been focused on using abundant biomass wastes as a feedstock for activated carbons. Oil-palm shell is a good candidate of raw materials for the production of activated carbon with a highly developed porosity and surface area because of the following reasons: the considerably high carbon content and relatively low price. It has been shown that the oil-palm shell has 55.7% of carbon content compared to oil-palm fibers (49.6%), coffee shells (50.3%), and sugar cane bagasse (53.1%) [4]. The relatively low price is because the raw materials are available as wastes, where they are free to obtain in small quantity or low price in huge quantity.

Oil-palm (*Elaeis guineensis *Jacq.) fruit is a major source of vegetable oil, which is extracted from its flesh and kernel. They are produced mainly in South East Asia (Malaysia, Indonesia, and Thailand), Africa (Nigeria and Cameroon), and several southern provinces of China. During palm oil processing, there are two major solid wastes generated, extracted flesh fiber (or called mesocarp) and seed shell (or called endocarp). In Malaysia, which is the largest palm oil producer in the world, about two million tons (dry weight) of oil-palm shell and one million ton of extracted oil-palm flesh fiber are generated annually. Normally, they are used as boiler fuels or building materials. Therefore better utilization of these cheap and abundant wastes is proposed to convert them into activated carbons.

The characterization of a carbon covers several properties, such as its complete pore size distribution, the quantitative analysis of the functional groups present at the surface, and the determination of the structural and pore heterogeneities of the solid by scanning electron microscopy. In mesopore (pore diameter of 2–50 nm) and macroporous (pore diameter > 50 nm) materials, the pores are thought to be filled with adsorbate by multilayer adsorption upon the internal pore surface and capillary condensation. Total internal surface area is therefore the primary factor controlling gas sorption in such materials.

Greater adsorption energies have been shown to exist in micropores (pore diameter < 2 nm) than in transitional pores of solids of similar composition. Micropores are believed to be filled by volume filling, as opposed to layer-by-layer adsorption on the internal surface of the pores. Micropore volume therefore appears to be the main control on gas sorption for microporous materials. The proportion of the total pore volume contributed by microporosity is thus an important parameter in evaluating the gas adsorption characteristics of a solid.

In the synthesis of activated carbon by physical activation, when a particular precursor is used, it is mainly the activation process that determines the classification of the final material. So it is important to conduct the activation under conditions that would produce an activated carbon with desirable textural and surface chemistry properties. From the survey, it was found that the effect of activation temperature on the porosity of the activated carbon varies with the raw material, and the experimental conditions cannot be extrapolated from one precursor material to another. Consider the case of activated carbon produced from a raw material of used tires, and activated with a mixture of steam and carbon-dioxide. The BET surface area of the activated carbon increased with activation time reaching a maximum of 432 m^2^/g after 120–150 min at a temperature of 970°C and a burn-off level of 60–65 weight % [3].

However, at temperatures above 900°C, the macroporous volume decreased slightly with increasing burn-off. Guo and Lua [2] produced activated carbons which were prepared from oil-palm shell and they showed that increasing the activation temperature from 773 to 1173 K leads to a progressive increase in the BET surface area; however, the maximum surface area obtained was 1366 m^2^/g, and all of samples they investigated were using holding time before activation in range of 60 to 120 min. In another study on oil-palm shell, Daud and Ali [5] found that within the temperature range of 800 to 900°C, an increase in the activation temperatures had no significant effect on the mesopore and macropore development. In general, the activation process is usually carried out in the presence of a suitable oxidizing agent such as steam, carbon-dioxide, air, or oxygen. For steam and carbon dioxide activation, the temperature is generally in the region of 800 to 1000°C, while steam activation starts in the temperature region of 700 to 750°C. However, for oxygen activation, a lower temperature of 300 to 500°C is usually employed to avoid combustion. The activation process, together with the intrinsic nature of the precursors, strongly determines the characteristics of the resulting activated carbons [5, 6].

According to the preceding discussion, this study is distinctive from previous work in a number of ways. First, it involves the development and modification of physical activation activated carbon-procedure by using a new basic medium ways to produce activated carbon with a high surface area and controlled pore structure. Second, it also includes a study of the effect of temperature on the porosity, chemical structure, and carbon surfaces of the activated carbons. 

However, same as previous existing studies in activated carbon characterization, nitrogen adsorption at 77 K is employed to verify the effect of the pore structure on the adsorption properties.

## 2. Experiment

### 2.1. Raw Material

The selection of appropriate raw material for the preparation of porous carbon, factors such as high carbon and low inorganic (i.e., low ash) content, high density of the precursor, and sufficient volatile content are taken into considerations [4]. The raw oil-palm shells were obtained from an oil-palm mill in Batu Pahat Johor, Malaysia. The experiments were conducted in the Thermodynamic Laboratory of Faculty of Mechanical Engineering, Universiti Teknikal Malaysia (UTeM), Melaka. The production of carbon adsorbents involved carbonization and physical activation process using an experimental rig, also known as activated carbon reactor.

### 2.2. Carbonization

**Figure 1:**
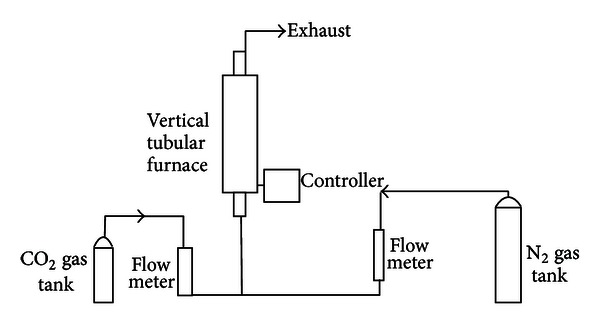
Schematic diagram of the experimental setup.

The raw oil-palm shell was first dried at 110°C to reduce the moisture content. The dried shell was then ground and sieved. Size fractions of 2.0–2.8 mm for shell were used.  Figure 1 shows the experimental setup for carbonization process that both pyrolysis and activation were carried out in a stainless-steel reactor having 550 mm length and 38 mm internal diameter, placed in a vertical tube furnace. About 50 g of the materials were placed on a metal mesh in the reactor. During pyrolysis, purified nitrogen at a flow rate of 150 cm^3^/min was used as purge gas. The furnace temperature was increased from room temperature to 500–700°C. The resulting chars were then directly activated based on its carbonization temperature (500–700°C) for 60 minutes under a CO_2_ flush.

### 2.3. Activation

The next stage was to activate the samples using carbon dioxide at a flow rate of 150 mL/min. The activation temperature was varied between 500 and 700°C with a heating rate of 10°C/min and an activation time of one hour. The carbonization and activation temperatures used in this study were lower than those used in the activated carbon industry where generally temperatures of 1000°C or more are employed. The use of lower temperatures agrees with past studies using lignocellulosic precursors [2]. Lower temperatures require less energy and help to reduce potential production costs. For convenience of discussion, the samples are labeled as in Table 1.

### 2.4. Characterization

The porous structure of each sample of the activated carbon was determined by nitrogen adsorption/desorption at 77 K using a surface analyzer Quantachrome, Autosorb-1c apparatus. Each sample was first degassed at 110°C for 12 hours under vacuum, and then the isotherm was measured in the relative pressure range from 0.01 to 0.99. The BET analysis was performed for relative pressures between 0.06 and 0.2. The total pore volume is calculated from the amount of vapor adsorbed at a relative pressure of 0.975. Following the IUPAC nomenclature, the pore sizes 2 nm and 50 nm are adopted as the micro-meso and meso-macro boundaries, respectively. The Barrett-Joyner-Halenda (BJH), Horvath-Kawazoe (HK), and density functional theory (DFT) methods are used here to investigate the micropore size distribution. The micropore volume is obtained from the amount of nitrogen adsorbed at a relative pressure of 0.2. The mesopore volume is calculated by subtracting the amount adsorbed at a relative pressure of 0.2 from that adsorbed at a relative pressure of 0.99. The DFT and slit-shaped pore models are then used to determine the mesopore size distribution. Finally, the average pore diameter is calculated from the ratio between micropore volume and surface area. A scanning electron microscope, Cambridge Instruments S360, was used to observe the presence of porosities and microporosities of the samples.

## 3. Results and Discussion

### 3.1. Surface Area and Pore Volume

**Figure 2:**
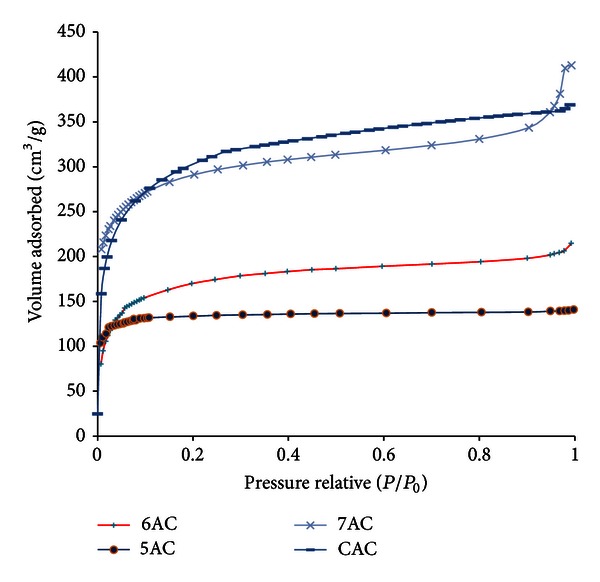
Results of adsorption isotherms of all the samples.

Figure 2 shows the adsorption isotherms which are obtained from nitrogen adsorption/desorption at 77 K using a surface analyzer. The results indicate a typical Type I according to IUPAC classification reflecting the domination of micropores in the pore structure. These are characteristic of microporous solids having a small external surface area, the limiting uptake being governed by the accessible micropore volume rather than by the internal surface area. In order to compare tailored activated carbon with the commercialized activated carbon, commercial activated carbon Norit AC was used and was provided by Esco Singapore Pte. Ltd. in the form of cylindrical extruders with the diameter of approximately 5 mm. This commercial activated carbon has been successfully characterized by Guan et al. [8].

At low pyrolysis temperature activated carbon in Figure 2, one can clearly see that the initial step region is abruptly followed by a plateau indicating that the adsorption has virtually stopped because multilayer of adsorbate cannot be formed due to close proximity of the pore wall. It also implies near absence of mesopore and macropore inside the material. Further increase in the temperature through activation process has widened the pores as well as increased the volume of micropores. This observation was also reported by González et al. [9] in steam and carbon dioxide activation of olive stone chars. The effect can be seen by the increase in the amount of gas adsorbed and the slope of the isotherm changes from a sharp knee at 500°C to a rounded knee with a discernible slope at high pressure region for higher temperature. This marginal increase in the volume adsorbed at the relative pressure range of 0.35–0.95 at high pyrolysis temperature could be attributed to the emergence of mesopores as a result of pore widening mechanism. More detailed discussion on pore development during activation process has been highlighted by Rodriguez-Reinoso [10]. The volumes were estimated from adsorption data by applying the Dubinin-Raduskevich equation over a range of relative pressure from 0.05 to 0.3.

The results of BET surface area, total pore volume, and micropore volume from the nitrogen adsorption/desorption for all the samples are summarized in Table 2. It can be seen that the BET surface area and the pore volumes increase as the carbonization temperature increases. The mesopore volume is shown to increase with the temperature due to pore widening upon activation with CO_2_ flow as stated in [11]. Table 2 also summaries the comparison between the samples with no holding time from this study and the samples with 60 minutes holding time temperature [7]. The preparation of activated carbon in [7] is similar with this study in terms of the raw material, the procedure, the heating rate, the carbonization, and activation processes except the holding time temperature.

It clearly shows that the samples with no holding time temperature have greater surface area, total pore volume, and micropore volume than the samples with holding time temperature.

Furthermore, it can be seen that micropore volume increases significantly with the increase of activation temperature. When the temperature is increased, the burn-off percentage of the fixed carbon will be higher and this also leads to the production of micropores. However, at temperatures of more than 1000°C as in [6] it was observed otherwise, the micropore fraction decreased. Extensive thermal activation could lead to pore widening, whereby the micropores created during the carbonization process will be widened to mesopores and macropores. As a result, volume of mesopore and macropore fractions will increase. This condition indirectly decreases the micropore fraction.

### 3.2. Pore Size Distribution

**Figure 3:**
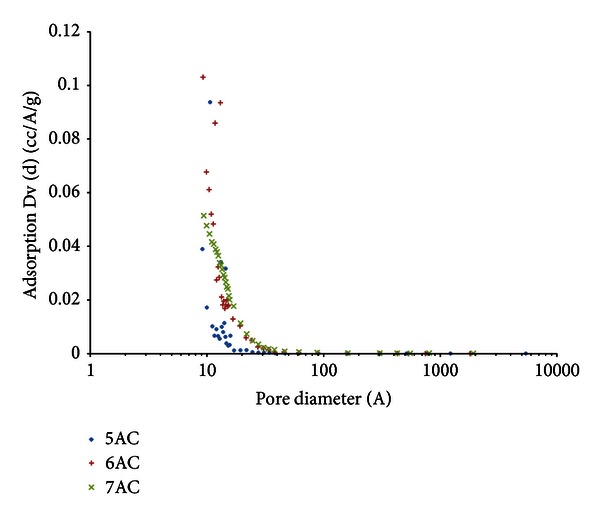
Results of PSD of palm shell activated carbon using BJH method.

Figure 3 shows the pore size distribution of the activated carbon using BJH method. It shows that there is a steep increase for amount of adsorption Dv (d) in the micropore region, indicating that micropores have made up a substantial volume of the total pore volume of the activated carbon.

This is further suggested that the prepared activated carbons were significantly made up of micropores. Similar pore size distributions were observed for all the other activated carbons obtained under different pyrolysis conditions.

**Figure 4:**
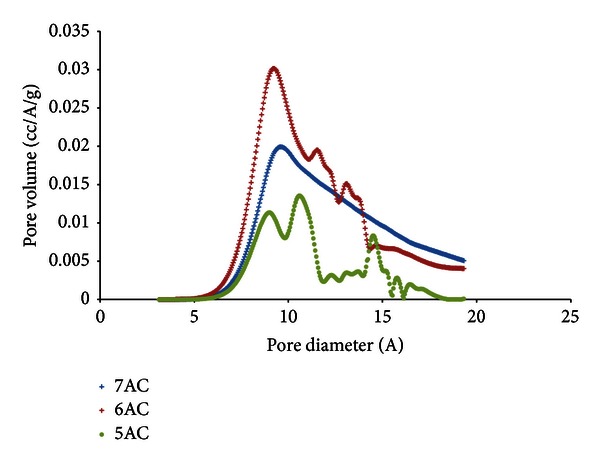
PSD of palm shell activated carbon using H-K method.

The micropore size distribution for the activated carbon according to the Horvath-Kawazoe method [12] is shown in Figure 4. From the differential pore volume plots, ultramicropores less than 7 Å are present, which contribute to selectivity of adsorption (molecular sieve effect). At activation temperature of 500°C, heterogeneous micropore size distribution is obtained with two different peaks at 8.6 Å and 11.4 Å while at 600°C the pore size distribution has shown more uniform size with peak at 9.8 Å. At 700°C, the activated carbon has one peak at 9.8 Å indicating that micropores made up a substantial volume of the total pore volume of the activated carbon.

**Figure 5:**
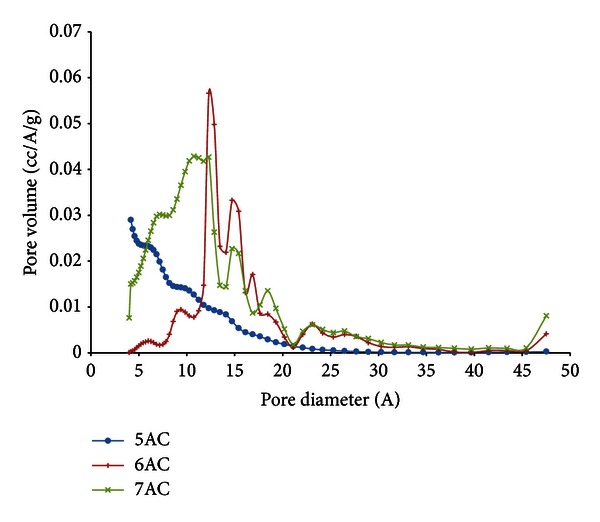
Results of PSD of palm shell activated carbon using DFT method.

**Figure 6:**
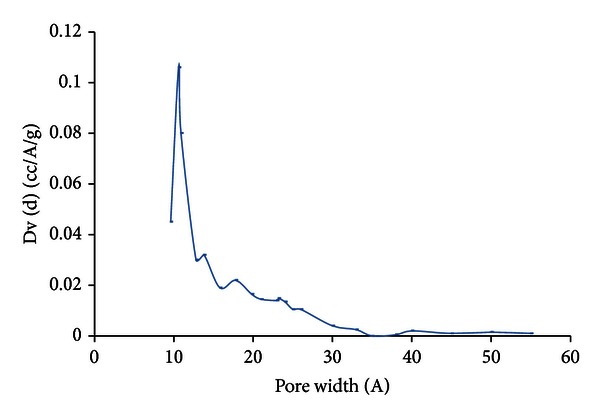
The PSD of the Norit AC derived from N_2_ isotherm at 77 K using DFT [8].

The PSD obtained by DFT analysis of the nitrogen adsorption isotherm is shown in Figure 5. It can be seen that the results are consistent with the isotherm trend discussed earlier. Based upon Figure 5, the activated carbon activated at 500°C contains 58.9% micropores, 29.9% mesopores, and 0.04% macropores while activated carbon prepared at 600°C contains 65.4% micropores and 34.5% mesopores. From Figure 6, it is shown that the Norit AC presents a narrow micropore peak around 11 Å with little mesoporosity.

### 3.3. Particle Morphology Characterization with Scanning Electron Microscopy Analysis

**Figure 7:**
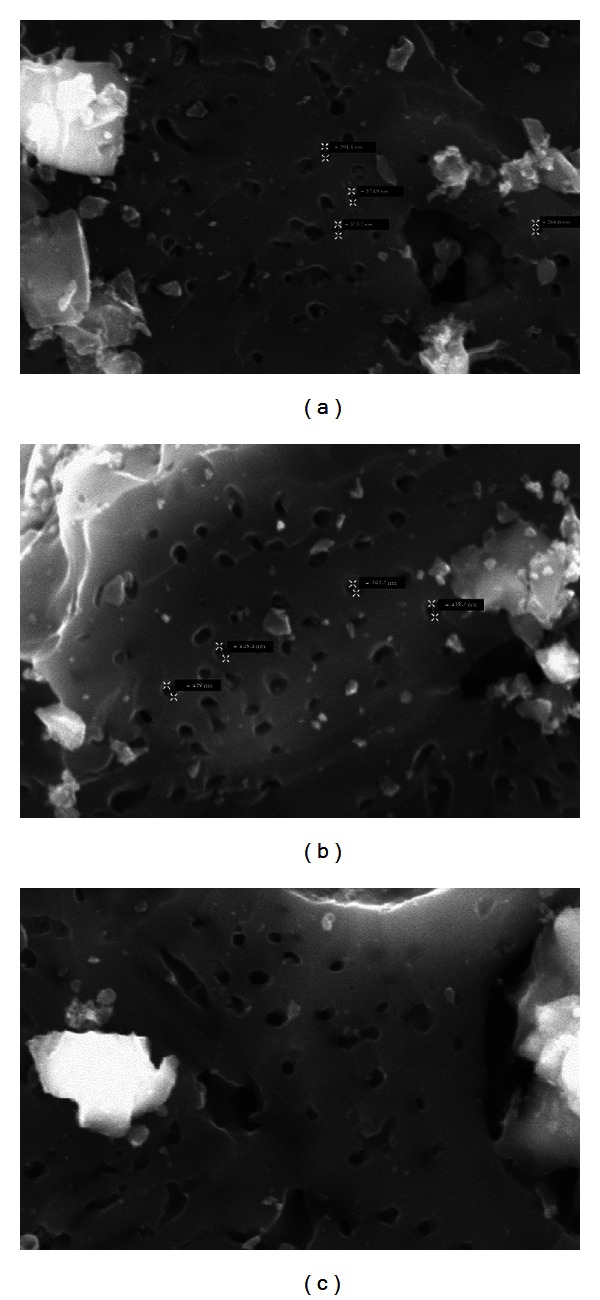
SEM photograph of oil-palm shell activated carbon prepared at (a) 500°C, (b) 600°C, and (c) 700°C.

The Scanning Electron Microscopy (SEM) analysis shows the surface condition of the fibrous activated carbon.  Figure 7 presents the micrographics of the activated carbon prepared at the selected temperatures.

The oil-palm shell activated carbon surface has a relatively smooth solid structure mainly void of pores but with occasional crevices. Similar to other SEM works [13, 14], the carbonized oil-palm shell surface was also covered with many globular silica bodies that contain sharp, conical agglomerations. From Figure 7, effect of pyrolysis temperature appears on the pore width and uniformness of the activated carbon can be observed. At all samples, some pores of carbon are covered with silica, while this silica may decrease the number of adsorbed particle due to the adsorbate that cannot enter the pore. This silica can be removed when higher CO_2_ flow is applied to the activated carbon preparation [15].

## 4. Conclusion

In this study, oil-palm shell-based activated carbons were produced successfully by modifying the CO_2_ activation method. Without holding temperature after pyrolysis, the experimental results show that relatively high pyrolysis temperature (700°C) is essential to remove volatile matters and develops rudimentary pore structures. As the activation temperature is increased from 500°C up to 700°C, the BET and micropore surface areas increase progressively since the activation process not only enlarges the pores created during the pyrolysis, but also generates some new pores. The pore size distributions also confirm the conversion of microporosity into mesoporosity or even macroporosity at a higher activation temperature. It is found that by shortening the preparation time, better product of activated carbons can be obtained.

## Figures and Tables

**Table 1:** List of label for the materials according to the process.

No.	Label	Meaning
1.	5AC	Carbonization and activation at 500°C
2.	6AC	Carbonization and activation at 600°C
3.	7AC	Carbonization and activation at 700°C
4.	CAC	Commercial activated carbon Norit
5.	A500*	Carbonization and activation at 500°C with 60 minutes holding time
6.	A600*	Carbonization and activation at 600°C with 60 minutes holding time
7.	A700*	Carbonization and activation at 700°C with 60 minutes holding time

*Results from [7].

**Table 2:** BET surface area and pore volume of palm shell activated carbon.

Sample	BET surface area (m^2^/gram)	Total pore volume (cm^3^/g)	Micropore volume (cm^3^/g)
5AC	521.5	0.215	0.217
6AC	631	0.314	0.288
7AC	905	0.569	0.449
CAC	860	0.55	0.48
A500*	317.357	0.14969	0.12376
A600*	369.318	0.17338	0.15288
A700*	369.595	0.17698	0.15549

*Results from [7].
